# The Use of Non-Apoptotic Sperm Selected by Magnetic Activated Cell Sorting (MACS) to Enhance Reproductive Outcomes: What the Evidence Says

**DOI:** 10.3390/biology13010030

**Published:** 2024-01-04

**Authors:** Nicolás Garrido, María Gil Juliá

**Affiliations:** IVIRMA Global Research Alliance, IVI Foundation, Andrology and Male Infertility Research Group, IIS La Fe Health Research Institute, Av. Fernando Abril Martorell, 106. Tower A, 1st Floor, 46026 Valencia, Spain; maria.gil@ivirma.com

**Keywords:** MACS, sperm, sperm selection, ICSI, cumulative live birth rate, pregnancy rate

## Abstract

**Simple Summary:**

Couples attending infertility clinics may suffer failed attempts and need consecutive treatments to achieve a healthy newborn, proving that there is room for improvement among the techniques we currently use. Selection of the right sperm—the most physiologically competent one—to be injected inside the egg among all available within the ejaculate is crucial to create good-quality embryos and increase cycle success rates. It is probably the step with the highest number of possibilities to choose from influencing the final outcomes. Apoptotic sperm, those undergoing programmed cell death, do not differ morphologically from healthy ones, their presence has been described as elevated in the ejaculates of infertile men, and they can fertilize the egg in both natural and artificial conception. In the latter, they can be unconsciously chosen by the embryologist and result in a poor-quality embryo that will stop developing or fail to implant. MACS enables the removal of apoptotic sperm from an ejaculate, thus leaving the non-apoptotic available to be microinjected. The existing literature on the topic provides conflicting evidence of variable quality that needs to be scrutinized and interpreted in order to define what is the benefit, if any, of using this technology and if this fits all infertile patients. We aim to provide clinicians and patients with a more accurate interpretation on how, when, and by how much, the use of MACS may impact their reproductive chances under an evidence-based medicine approach.

**Abstract:**

Sperm selection of the most competent sperm is a promising way to enhance reproductive outcomes. Apoptosis is the programmed cell death process to maintain tissue homeostasis, and MACS sperm selection of non-apoptotic cells enables the removal of apoptotic sperm from an ejaculate, thus leaving the non-apoptotic available to be microinjected, but given the associated costs of adding these sperm selection steps to the routine practice, there is a need for a careful examination of the literature available to answer questions such as who can benefit from this MACS, how significant this improvement is, and how robust the evidence and data available supporting this choice are. Thus, the aim of this narrative review was to objectively evaluate the available evidence regarding the potential benefits of the use of MACS. From the literature, there are controversial results since its implementation as an in vitro fertilization add-on, and this may be explained in part by the low quality of the evidence available, wrong designs, or even inadequate statistical analyses. We concluded that the benefits of adding MACS are unclear, and further methodologically sound research on specific populations is much needed before offering it clinically.

## 1. Infertility Prevalence and Male Contribution

Infertility, defined as the failure to conceive after 1 year of regular unprotected sexual intercourse, affects about 15% of couples at their reproductive age. Generally speaking, the male factor can be the attributable cause in about half of the cases either alone or together with subjacent female factors [1].

For both men and women, infertility is frequently a multifactorial condition. For men, it can range from the inability to produce sperm or deliver them via the ejaculate in the most severe cases, to milder presentations such as generating a lower number of sperm cells than those needed to procreate naturally during the above-mentioned time range. However, even if the concentration of sperm produced is adequate, spermatozoa may lack the physiological (genetic or biochemical) competence to generate embryos able to properly develop, implant, and result in a healthy child.

As several reports describe, male fertility has been significantly decreasing all over the world in recent decades, as reflected by the lower sperm counts compared with previous times and the historical trends on basic sperm analysis results with clinical consequences [2,3]. Unfortunately, we can only guess that molecular characteristics of sperm have also changed through the years, since no molecular data on this are available.

The reasons behind the infertile phenotype may include low functionality and adequacy of the genital tract, altered hormonal or biochemical conditions, among others, or the individual’s genetic cargo, environmental exposures, or a combination of two or more of these factors [4].

Spermatozoa are very specialized cells, with very particular functions, including displacement, being the carrier of the paternal genetic material and cytoplasmic components, recognition and fusion with the oocyte, and finally, production through the created zygote’s development, implantation and growth of a new human being [1,4].

To this end, competent sperm creation, processing and maturation through the progression of spermatogenesis need the orchestrated interaction of numerous genes and routes, which can be affected by a myriad of factors, both external and internal. Disturbing this delicate equilibrium may lead to the production of insufficient or incompetent sperm cells jeopardizing reproductive success at any of its steps [1,4] or by any via either natural or assisted conception. In the latter, these incompetent spermatozoa may be easily overlooked if their morphological appearance seems normal, unless we have the proper tools to identify them and act accordingly. Each sperm differs from others within the same cohort, both genetically and physiologically, and selecting the right sperm among all those present within an ejaculate may improve reproductive outcomes by generating better embryos.

Sperm cell apoptosis has been described as one of the causes of male factor infertility. A higher proportion of pro-apoptotic sperm has been observed in infertile men compared to fertile men. In these cases, eggs are fertilized with sperm with initiated programmed cell death pathways resulting in embryos with poor developmental potential that will frequently arrest [5,6,7,8]. Subsequently, the possibility of removing apoptotic sperm and enriching a semen sample with non-apoptotic sperm to then be used in assisted reproduction techniques (ART) and improve cycle outcomes has become of interest, although it has been technically available for decades through several technologies such as magnetic activated cell-sorting (MACS) [9].

This review elaborates on the pathophysiological mechanisms of sperm apoptosis, how these may affect embryo quality and ART outcomes, how to decrease the population of apoptotic sperm in order to increase reproductive chances, and if this intervention has any effect on the main outcome measures used in ART within the context of the quality of the evidence available so far.

## 2. Apoptosis: What Is It and How Is It Related with Male Infertility?

Apoptosis is a physiological process aiming to eliminate unwanted/unnecessary, damaged and excess cells among organs and tissues to maintain homeostasis and proper function. It can be triggered by external as well as internal environmental stimuli such as age, infection, oxidative stress, tissue development, etc., and has been linked also to hormonal activity, immune function, embryo development, among other physiological events [10]. This process actively prompts programmed cell death following different steps that have been well characterized in a number of cell types, which takes time and uses energy to be completed.

Malfunctioning apoptotic activity (excessive, insufficient or defective) has been linked to several pathological conditions in humans: neurodegenerative diseases, ischemic injury, autoimmune conditions and cancer, as well as male fertility, clearly suggesting that the ability to modulate this process holds therapeutic potential.

Generally speaking, there are a number of morphological, molecular and immunological characteristics that may be specific of different cell types [11] characterizing an apoptotic cell. These phenomena might be jeopardizing the reproductive capacity in males.

Although apoptotic mechanisms have not been fully described in human spermatozoa and their presentation differs from most somatic cells [12], caspases play a crucial role in this pathway: the initiating stimulus triggers death receptors such as the tumor necrosis factor receptor or CD45, which leads to the activation of the initiator caspase 8, located in the post-acrosomal region of the spermatozoon. Together with other adaptor proteins, it conforms the death-inducing signaling complex in early apoptosis. One of the manifestations of early apoptosis is the damage to the cell membrane and the consequent externalization of phosphatidylserine (PS) from the inner to the outer leaflet of the sperm membrane. Following a different route, caspase 9—located in the midpiece of the sperm cell—interacts with transducer proteins such as cytochrome c to initiate mitochondrial membrane dysfunction. In later stages of apoptosis, caspase 3 will produce the cleavage of structural proteins and the breakage of DNA strands [13]. More than two decades ago, Paasch and colleagues described an increase in active caspases and externalized PS in spermatozoa from infertile men compared to fertile men [7]. Unfortunately, sperm that have initiated apoptosis will experience these—and more—biochemical changes without necessarily exhibiting significant alterations to their morphology and motility, which allows them to escape programmed cell death and fertilize oocytes [14,15,16].

Subsequently, we can hypothesize that natural reproduction could be significantly harmed, since the possibility of having spermatozoa who already initiated the apoptotic process of fertilizing an oocyte is plausible, and the greater the extent of apoptotic sperm within an ejaculate, the bigger the risk of reproductive failure.

This situation can also occur using ART, since apoptotic sperm are present in semen samples with morphology and motility parameters below normality [12,17,18,19,20] cases with acrosome reaction defects and low fertilizing potential [21,22]—patients that would normally enroll in an infertility treatment due to male factor—and there is no morphological way to differentiate apoptotic from non-apoptotic sperm [23]. This introduces a significant risk in the IVF lab of blindly picking sperm who already initiated the apoptotic process to be either used in intrauterine insemination treatments or microinjected into the oocytes, negatively impacting reproductive outcomes. Hence, there is growing interest in the andrology field to non-invasively improve sperm selection, eliminating the apoptotic fraction of semen samples to ensure they are enriched with competent spermatozoa to perform ART safely.

## 3. Selecting the Best Spermatozoa—MACS

As we have previously discussed, the selection of the appropriate/most apt sperm can be crucial to the success of an IVF cycle. Basing this selection on specific molecular features is, therefore, extremely interesting in order to potentially improve overall outcomes. Despite the development of new technologies for the assessment, preparation and selection of sperm in ART, their progress and implementation in the clinic has been frustrating thus far. Moreover, the literature shows very inconsistent conclusions, since the methods and results reported have provided highly variable information about the benefits in improving outcomes after ART [1,4].

Even though previous research on sperm selection for ICSI provides embryologists and andrologists with precise morphological criteria to select spermatozoa with the highest probabilities of success [24], this approach to sperm evaluation neglects both the molecular and genetic competency and uniqueness of each spermatozoon. Choosing an inadequate fertilizing sperm can lead to fertilization failure, incorrect embryo development, failed implantation, or miscarriage. Thus, sperm selection is crucial to ensure that the oocyte is correctly fertilized by the most competent sperm [25].

In order to select particular cells from a heterogeneous population, there is a need for well-characterized and specific markers of their identity [26], characteristic to that specific cell. These biomarkers would then be used to separate the target cells from the rest of the population using a technique that leaves cell viability and functionality intact. Most of these separation approaches rely on fluorescent labelling, either using an antibody that specifically recognizes the target marker conjugated with a fluorescent agent or the expression of a genetically engineered fluorescent protein exclusively in the target cell type [27,28,29]. However, this approach is limited by the antibodies’ availability, cross-reactivity to other targets, and unspecific labelling [30,31,32]. Moreover, typically only surface proteins can be targeted, since antibodies and other recognition molecules are generally unable to cross the cell membrane. Another potential issue is the undesired digestion of surface proteins—potential membrane markers—by enzymes used in the dissociation procedure [33,34]. Although extending the recovery time after dissociation would allow for the re-synthesis of surface markers, it could disturb the expression profiles [35,36]. Another approach uses electrophoresis to sort the target cells according to their membrane electrical potential [37,38,39], but this technique is destructive, as some of the previously described ones are, preventing us from using the assessed sperm for ART.

Magnetic-activated cell sorting (MACS) is a non-destructive cell separation technique that allows for the retention of apoptotic sperm cells expressing phosphatidylserine in their external membrane inside a column [22,40,41]. During early apoptosis, damaged sperm would externalize the phospholipid phosphatidylserine (PS), normally present on the inner leaflet of the sperm plasma membrane, to the outer leaflet. This technique relies on paramagnetic beads conjugated with annexin-V to recognize and bind to the externalized PS, for which it has high affinity [42,43]. When a strong magnetic field is applied, the fraction that is retained is identified as pro-apoptotic annexin V-positive while the fraction that corresponds to non-apoptotic annexin V-negative spermatozoa elutes through the column [44,45,46]. The eluted sample is enriched with non-apoptotic sperm, ready to be used in ART [47]. Said and colleagues demonstrated the effectiveness of this non-invasive method to select non-apoptotic spermatozoa in samples with high average sperm apoptosis in the ejaculate [45,48].

## 4. Basic Studies: Using MACS to Improve Sperm Sample Quality

Despite not being performed routinely in the clinic, MACS is frequently suggested to patients with a high spermatic DNA fragmentation index, more than two unexplained ICSI failures and, in certain cases, more than two miscarriages with an unknown female cause [49].

MACS combined with density gradient centrifugation (DGC) has been associated with a higher recovery of sperm with progressive motility (68%) when compared to neat ejaculate (39%) (*p* < 0.05), as well as a lower DNA fragmentation index (4% MACS-DGC versus 24% in the reference; *p* > 0.05) [17] and an increase in the percentage of sperm with normal morphology (2.44% in the MACS separation group versus 0.92% in the raw sample group; *p* < 0.01) [50].

In some studies, sperm selection via MACS showed a reduction in spermatic DNA fragmentation (fDNA) when compared to the neat ejaculate from asthenoteratozoospermic, teratozoospermic and normozoospermic men [51,52]. However, one of these studies reported that the reduction in spermatic fDNA was not complete and not significant in all patients, only when samples had an initial fragmentation index ≥30% (7.1% after MACS versus 41.4% in the neat ejaculate) [53]. Another study reported no significant improvement of sperm morphology, motility, fDNA or markers of fertilization capability Izumo-1 and PLC-ζ comparing MACS combined with swim-up or DGC against controls [54].

Aside from the effect the use of MACS may have on improving sperm parameters, there is a lack of agreement on the impact the technique has on ART cycle outcomes, as highlighted by recent meta-analyses [55,56].

## 5. Uses of MACS of Non-Apoptotic Sperm: Clinical Studies

### 5.1. Case Reports

Although the first report on the clinical use of MACS dates from 2008 [50], to present the literature available by quality of evidence, we will start describing the initial case reports available.

Rawe et al., in 2010 [57] reported a case where a 37-year-old woman with 4 years of primary infertility due to male factor (38-year-old male who had previously been surgically treated for bilateral varicocele), normal day-3 FSH, LH and estradiol levels, normal karyotype on both, six cycles of failed intrauterine inseminations, and a 31% sperm fDNA index measured using terminal deoxynucleotidyl transferase-mediated dUDP nick-end labelling (TUNEL) assay, where antioxidants were given for 3 months and a standard ICSI cycle was performed, fertilization rates (FR) were 45%, with bad quality embryos, and two of them were transferred on D3 filing to achieve pregnancy. In the subsequent cycle, detection of sperm apoptosis (fDNA by TUNEL and cleaved caspase 3 Asp175 by immunocytochemistry), respectively, showed 25% of sperm with fDNA and cleaved caspase 3 in 8% of spermatozoa. Upon confirmation of apoptosis, they used MACS before ICSI and found a significant reduction in fDNA from 25% to 10%. Cleaved caspase 3 was also reduced from 8% to 7% after MACS, but this difference was not significant. FR increased to 60%, obtaining good-quality day-2 and -3 embryos, two of which were transferred and resulted in a healthy newborn.

Another case series report, from Polak de Fried, also in 2010 [58], showed two more cases: the first one was in a premature ovarian failure patient undergoing oocyte donation, with the male showing asthenoteratozoospermia and abnormal fDNA (TUNEL 30%). They performed MACS prior to ICSI, injecting six metaphase II (MII) permitting two embryos to be transferred with an additional one frozen, resulting in an ongoing pregnancy. The second case involved a couple with more than 4 years of primary infertility and recent ICSI failure. The male was teratozoospermic and presented elevated active caspase-3 (16%). They repeated ICSI on nine MII oocytes with MACS-selected spermatozoa, showing total fertilization, two embryos transferred, and an additional blastocyst cryopreserved, resulting in a twin pregnancy.

Herrero et al., in 2013 [59] presented a case from a couple in which the 34-year-old male partner was a survivor of non-Hodgkin’s lymphoma. He cryopreserved sperm at 28, prior to chemotherapy. The couple had a history of recurrent IVF/ICSI failure using those frozen samples. When analyzed, the post-annexin V-MACS sperm sample showed a significant reduction in fragmented DNA (from ≈75% to <60%) compared with the untreated sample. Eight MII oocytes were collected and injected. As a result, four of them were fertilized and two day-3 embryos were transferred, and healthy twins were born.

A summary of these studies’ main findings is shown in Table 1.

Although used successfully in particular cases, these reports at that time were not definitive proof of the benefit of using MACS in specific cases of high DNA fragmentation or active caspases versus the standard procedures, due to the lack of a control group to compare with and the extremely limited sample size, resulting in a very low overall level of evidence.

### 5.2. Non-Randomized Trials: Superiority versus Standard Processing

#### 5.2.1. Retrospective Studies

In their paper published in 2017, Sánchez Martín et al. [35] retrospectively evaluated whether reproductive outcomes can be improved in couples exhibiting high sperm DNA fragmentation levels (with ≥30% sperm DNA in the ejaculate) by non-apoptotic MACS sperm selection combined with prior DGC, compared with DGC alone. A total of 305 couples were included, 216 with autologous ICSI (AUTO-ICSI), and 89 in oocyte donor ICSI (DONOR-ICSI). The authors found similar live birth rates (LBR) on both cases of >30–50% and cases of >50% sperm DNA fragmentation, and the main finding was the lack of any miscarriage in either cohort of patients following MACS (Table 1).

Pacheco and colleagues [60] retrospectively investigated the effect of MACS on cycle outcomes of patients with high levels of spermatic fDNA. A total of 724 couples undergoing ICSI were split into two groups: the control group, in which only DGC was used to process 358 samples; and the study group, in which 366 samples were processed using DGC followed by MACS. Interestingly, outcomes were subanalyzed into cycles of preimplantational genetic testing for aneuploidy (PGT-A), autologous oocyte cycles and oocyte donation cycles, adding novel information to the available literature. A significantly lower miscarriage rate was observed in the DGC-MACS group in autologous ICSI cycles (11.3% vs. 25.5% in the controls; *p* = 0.005), higher PR in oocyte donation cycles (69.3% vs. 53.9%; *p* = 0.013), and a significant increase in LBR in both autologous (40.9% vs. 24.6%; *p* = 0.03) and oocyte donation cycles (51.8% vs. 29.4%; *p* = 0.03) (Table 1). They concluded that MACS can be effectively used to improve reproductive outcomes in specific subsets of couples undergoing ART, depending on the origin of the oocytes undergoing ART. Again, this promising approach, with a noticeable sample size in a retrospective analysis, needs to be statistically controlled for relevant factors potentially influencing outcomes, and ideally, considering the additional contribution of embryos transferred following frozen/thawed cycles.

#### 5.2.2. Prospective Studies

The first prospective comparative cohort study was published by the Turkish group of Dirican in 2008. Two groups of oligospermic men undergoing ICSI were compared: the study group formed of 122 couples using non-apoptotic MACS-selected spermatozoa and the control group with 74 couples using only DGC for sperm processing [50]. The results confirmed that the percentage of sperm with normal morphology after MACS selection was improved from around 1% to 2.4%. In terms of cycle outcomes, the authors reported a slight increase in the clinical pregnancy rate (PR) in the MACS group (36.49% to 48.36%, *p* = 0.052), although the difference was not statistically significant, and neither was the implantation rate (IR) (21.9% in the study group versus 19.3% in the controls, p not specified). Although prospective, this study had no statement of randomization, nor did it control for any confounding parameters in the statistical analysis. Intermediate milestone findings such as significant differences in biochemical pregnancy and cleavage rate (CR) should not hide the most important result, which is live birth, for which there no information has been collected. Additionally, the contribution from surplus embryos obtained from these cycles has not been quantified.

Sheikhi et al., in 2013 [36], evaluated whether elimination of apoptotic spermatozoa for couples with unexplained infertility (UI) could increase the likelihood of pregnancy by ICSI. The study included 74 couples: 37 of them had their sperm sample processed by DGC prior to ICSI—this group was used as a reference—and the other 37 had their sperm sample processed by DGC followed by MACS (DGC-MACS). They observed that FR was significantly higher in the DGC-MACS group compared to the reference (73.41% vs. 61.11%; *p* = 0.03), as well as the day-3 eight blastomere non-fragmented embryos per oocyte injected (45.05% DGC-MACS vs. 34.16% DGC only; *p* = 0.049). Though not statistically significant, there was a slight difference in pregnancy and live birth rates between the DGC-MACS and reference groups (43.24% vs. 40.5% and 35.11% vs. 27% respectively). Similar to the previously discussed study, couples were not randomized, nor did the statistical analysis include multivariant approaches controlling for the main variables potentially influencing the results. Moreover, only using the first embryo transfer (ET) to compare between groups could lead to a negative selection bias, since the entire cohort’s contribution produced from each insemination has not been evaluated. The fact that the remaining frozen embryos present separate opportunities to achieve a different outcome in each group has not been properly addressed.

García-Ferreyra, also in 2013 [61] reported high pregnancy and IR obtained using MACS in 57 patients with high levels of spermatic fDNA when compared to 77 control patients with normal fDNA whose spermatozoa were elected according to classic morphological characteristics. They reported that the FR, CR, embryo quality, PR, IR and miscarriage rate (MR) were similar between groups. However, these results should be assessed critically since the patient groups are not correctly designed. The appropriate comparison would have been to compare the MACS group to a control conformed by patients with high fDNA whose sperm were selected by classic morphological criteria.

Stimpfel and collaborators, in 2018 [62], aimed to evaluate the effect of MACS for sperm selection prior to ICSI for couples with teratozoospermic men and women with good prognoses. They enrolled 26 couples undergoing ICSI following a sibling oocyte design; half the oocytes were inseminated using sperm prepared with DGC (reference), and the other half were microinjected with sperm processed through DGC-MACS (study group). How the oocytes were assigned to each treatment group was not described. They found that while the overall percentage of morphologically normal spermatozoa was comparable, there was a significantly higher rate of abnormal tailed sperm in the study group compared to the reference. The authors attributed this to the selection procedure. FR, embryo quality, PR, and delivery rates were compared between both groups. After dividing patients according to female age, the subanalysis revealed that the study group showed a higher rate of good-quality blastocysts in women aged ≥31 compared to the reference (75.0% vs. 33.3%; *p* = 0.028).

In 2022, Salehi Novin and colleagues [63] examined seminal parameters, fDNA index (DFI), and PLCz1 expression levels in 60 samples with a starting DFI > 30%, pre- and post-processing by either DGC (reference group) or MACS-DGC (study group). The oocytes retrieved from a given patient were assigned at random to the reference group (n = 86 oocytes) or study group (n = 102), and they were injected with sperm processed accordingly during ICSI. PLCz1 expression was considerably higher in the study group compared to the reference. Although the FR values were comparable between groups (77.8% DGC only vs. 79.3% MACS-DGC; *p* = 0.445), the study group showed a higher rate of top-quality day-3 embryos (63.23 ± 0.44 vs. 85.00 ± 0.57; *p* = 0.038) and a higher blastocyst rate (48.00% vs. 69.69%; *p* < 0.001).

Sperm processing using MACS-DGC could potentially enrich the sample with sperm expressing high levels of PLCz1 by discarding pro-apoptotic cells. This would, in turn, optimize sperm selection for patients with high fDNA, improving the development of the resulting embryos.

Generally speaking, when it comes to proving the utility of MACS, the retrospective analyses typically lack sufficient robustness due to several methodological limitations such as inadequate sample sizes, lack of control of potential confounders, and not considering the full contribution of each sperm selection methodology given that surplus frozen embryos’ transferences are not included within the analyses presented, which is a similar situation compared with non-randomized prospective studies. Although prospectively acquired data tend to be more reliable, the methodological limitations, if not taken into account, are comparable to those from retrospective research.

The summary of these studies’ main results is also shown in Table 1.

### 5.3. Prospective Randomized Controlled Trials

Randomized controlled trials provide the best evidence for supporting or rejecting the contribution of any intervention on a defined outcome. The power of this methodological design to compare new interventions versus standard resides in the fact that randomization of sufficient patients to control groups, where standard treatments are provided, or to the studied intervention, will theoretically randomly distribute all known (those that have been already related with the main outcome measured, and are registered within the trial) and unknown (those from whose there is no information about its potential relationship with the main outcome, and then are not measured during the trial) factors, thus minimizing any potential influence on the results.

Romany and colleagues in 2014 [64] studied the effect of removing apoptotic sperm cells by MACS in samples from unselected male patients on LBR after ICSI. To homogenize the female factor, they only included ovum donation cycles in a prospective, randomized, triple-blinded, and controlled study with a total of 237 infertile couples. Semen specimens prepared by swim-up and swim-up followed by MACS were used.

When comparing the MACS group to the control, FR was 75.3% (95%CI, 71.6–78.9) versus 72.1% (68.6–75.7); the rate of day-2 good-quality embryos was 53.7% (50.3–57.1) versus 51.8% (48.3–55.3), and on day 3, 54.2% (50.7–57.6) versus 48.9% (45.3–52.4); IR of 42.2% (33.8–48.1) versus 40.1% (34.8–49.6); positive beta-hCG tests of 63.2% (54.7–71.6) versus 68.6% (60.2–76.9), and LBR of 48.4% (39.6–57.1) versus 56.4% (47.3–65.5). All outcomes were statistically comparable between groups, concluding the lack of utility of this sperm selection method for unselected males undergoing ICSI oocyte donation cycles (Table 2). Although some cases were lost during the follow-up, the number of recruited patients may, to some extent, maintain the properties of randomization.

In another RCT, Troya and colleagues [65] included 47 women in the hyaluronic-acid-ICSI group—otherwise known as physiologic ICSI or PICSI for short—33 women in the MACS group, and 55 women in the ICSI control group. All male partners had normal semen parameters in accordance with WHO 2010 criteria. They found similar FR and an average number of day-3 embryos and number of frozen blastocysts for the ICSI, PICSI and MACS groups. Although there was a statistically significant difference between the groups in clinical PRs: 58.1% in the MACS group, 40.4% in the PICSI group and 27.3% in the controls (*p* = 0.019, this was not the case in biochemical PR and pregnancy loss rate. DFI was assessed using a sperm chromatin dispersion test (SCD; specifically, the Halosperm kit) in both the apoptotic and non-apoptotic sperm fractions of 17 samples after performing MACS. The authors observed a statistically significant decrease in DFI in the non-apoptotic fraction after MACS (*p* = 0.000), thus concluding that sperm processing through MACS successfully enriches the sample with low DF spermatozoa, which improves clinical pregnancy rates for infertile couples (Table 2). A subsequent Cochrane review paper cataloged this work as very low quality for the evidence obtained, due to several methodological issues [56], i.e., incomplete outcome data (attrition bias), exemplified when three MACS cases were removed when analyzing the PR since they did not have an ET, and uncertain proper randomization yielding unbalanced groups, suggesting a risk on the random sequence generation (selection bias). Additionally, the lack of a description of how the sample size was calculated makes these results controversial.

In 2019, Ziarati and collaborators [66] performed a prospective randomized trial in which 62 couples with male factor infertility—defined as having at least two basic sperm parameters below the WHO 2010 standards—were allocated into the reference group (sperm was processed using only DGC; n = 33) and the study group (processing performed by MACS-DGC; n = 29). Although there was no statistically significant difference in FR between the groups, the rates of high-quality or A-score embryos (35.85% ± 06.58 vs. 20.00% ± 03.93; *p* = 0.04), PR (54.54% vs. 24.24%; *p* = 0.01) and IR (36.3% vs. 15.7%; *p* = 0.02) were significantly higher in the MACS–DGC group compared to DGC alone (Table 2). Nevertheless, their conclusions are weakened by a number of issues, namely the limited number of patients included, the method of randomization, estimation of the sample size, absence of blinding, the lack of information on allocation concealment and having data not analyzed by intention-to-treat (if the patients lost patients reported per group—11 in the MACS and 7 in the controls—had been included in the analysis, the statistical significant would have vanished).

Hasanen and colleagues, in 2020 [67] compared PICSI vs. MACS in a prospective randomized trial in which 413 ICSI cases with abnormal spermatic fDNA (> 20.3%) measured by TUNEL assay were randomized into each of the study groups. They found no significant differences on pre-implantation embryological data, IR, clinical and ongoing PR. In a consequent subanalysis, patients were divided accorded to the female age. Women aged <30 had a higher rate of good-quality blastocysts when the sperm sample was processed by MACS before ICSI compared to PICSI (71.1% ± 25.4 vs. 62.9% ± 24.7; *p* = 0.03), an increased clinical PR (73.9% vs. 58.3%; *p* = 0.03) as well as ongoing PR (69.5% vs. 51.3%; *p* = 0.01). These differences, however, were not significant in the 30–35-year-old female group (Table 2). The authors concluded that both PICSI and MACS were successful in the improvement of outcomes in younger women and men with abnormal fDNA.

The only report on donated sperm comes from González-Ravina et al., in 2022 [68]. Since cryopreservation of sperm in donors is mandatory due to the regulations involved to avoid the window period for some infectious diseases [69] and the freezing–thawing process can be detrimental to the survival and functionality of spermatozoa, the authors evaluated the effect of processing donor samples for intrauterine insemination (IUI) by MACS on reproductive outcomes. This multicentric prospective randomized study analyzed clinical outcomes of 181 donor IUI treatments, with MACS performed after DGC in 90 thawed semen donor samples, and only DGC in 91. Their results show comparable PR (26.7% vs. 26.4%; *p* = 0.96), LBR (58.3% vs. 50.0%; *p* = 0.56) and MR (41.7% vs. 50.0%; *p* = 0.56) between the two groups (Table 2).

### 5.4. Meta-Analyses

In 2013, Gil et al. [55] performed a systematic review and meta-analysis of only prospective randomized trials on this topic to determine whether the use of MACS improves success rates in couples undergoing ART. They identified 5 studies comprising 499 patients and found a statistically significant difference in PR when compared with sperm preparation by DGC or swim-up (RR = 1.50, 95% CI 1.14–1.98), while IR (RR = 1.03, 95% CI 0.80–1.31) and MR (RR = 2.00, 95% CI 0.19–20.90) remained statistically comparable.

This information was further scrutinized and updated in a Cochrane Database of Systematic Reviews for Advanced sperm selection techniques for assisted reproduction (Lepine et al., 2019). This report identified one RCT comparing LBR between MACS prior to ICSI to standard ICSI, three documenting clinical PR, and two disclosing MR. They determined that the quality of evidence was very low, thus concluding that, generally speaking, it is uncertain whether MACS improves either LBR or clinical PR, or if MACS reduces MR per woman or clinical pregnancy.

One RCT examined in this report compared MACS to PICSI for sperm selection prior to ICSI. However, it did not account for LBR, and it did not clarify whether MACS had an effect on the MR per patient (RR 1.52, 95%CI (0.10–23.35), n = 78 women) or per clinical pregnancy (RR 1.06, 95%CI (0.07–15.64), n = 37 women), or its effect on clinical PR (RR 1.44, 95%CI (0.91–2.27), n = 78 women). The Cochrane review considered the quality of the evidence in this study ‘very low’.

The authors concluded that the MACS effect on LBR, MR or PR is uncertain, needing more high-quality and well-designed studies, as well as the addition of the remaining data from the ongoing studies to the analyses, so clinical application should be kept on hold (Table 2).

## 6. The Use of CLBR as an Improved Measure of Success

The way in which ART cycle outcomes are expressed is fundamental to give patients and clinicians the most accurate and intelligible estimation of success. Embryo quality, IR or PR per ET are often used to evaluate the effectiveness of treatments. Nonetheless, intermediate parameters such as these do not always undoubtedly reflect the likelihood of a couple to obtain a healthy newborn for each ART treatment started. LBR per ET is the most frequently used metric to measure reproductive outcomes [70], often being insufficient or incorrect. There are cases where using LBR is biasing results to a negative finding or smoothing the actual impact, considering only the contribution of the best embryos obtained—the ones chosen to transfer, not that of the remaining embryos within the same cohort. The cumulative live birth rate (CLBR) controls this bias to some extent since it accounts for the number of ET, embryos transferred, or oocytes required so that a couple undergoing a particular ART can achieve a live birth [71,72,73]. This measurement considers the influence of each microinjected spermatozoon on the final reproductive outcome.

Accordingly, Gil Juliá et al. [74] retrospectively evaluated the effect of MACS sperm processing in autologous ICSI cycles on the CLBR per ET, per embryo replaced and metaphase II (MII) oocyte used, providing a more truthful view of the impact of the intervention [71,72,73]. Patients were divided according to the method with which their semen sample was processed before ICSI into standard capacitation via washing, DGC or swim-up (46,807 patients) versus those that added a MACS step (1779 patients). The authors concluded that there were no statistically significant differences between the groups in clinical PR (38.48% in the controls vs. 39.68% in the MACS group, *p* = 0.1956), ongoing PR (31.80% vs. 32.41%; *p* = 0.4904) and LBR (29.20% vs. 29.30%; *p* = 0.9154) per transfer and LBR per cycle (37.40% vs. 38.82%; *p* = 0.1907). The differences between groups in LBR per transfer and LBR per cycle remained non-significant after adjusting according to the female patient’s age and BMI, age of the male patient, last recorded endometrial lining in mm, male factor, transfer at the blastocyst stage and whether the cycle included PGT-A [74]. The difference in CLBR between the groups was only statistically significant when measured per embryo transferred. Ultimately, the results suggest that, although the CLBR per embryo replaced was slightly higher in the MACS group, unselected males undergoing autologous ICSI cycles do not benefit from non-apoptotic sperm selection (Table 3).

The use of autologous—patient’s own—oocytes is an additional source of variability, already in combination with patient and cycle heterogeneity within the study population. Oocyte donation has been proven to be a good model to test the male contribution to reproductive success [75]. In order to isolate the sperm contribution to reproductive success as much as possible, Gil Juliá et al. performed a subsequent retrospective analysis focusing on ICSI cycles using a donor oocyte, which allowed for the standardization of the oocyte quality factor homogenizing the population to strictly selected healthy young donors with optimal ovarian reserve [49]. With data from 37,269 ICSI cycles, the CLBR in the MACS group after one and after four embryos replaced were 27.1 and 81.6%, while the reference group—standard sperm processing—showed a CLBR of 19.6% and 78.5%, respectively, and the comparison between both Kaplan–Meier curves reported the differences as statistically significant (*p* < 0.0001). When considering MII oocytes injected, the CLBR in the MACS group was 4.2% after five oocytes and 75.5% after fifteen, whereas these were 7.8% and 78.3%, respectively, in the reference group. The Mantel–Cox comparison also showed statistical significance in the differences between the groups in this case (*p* < 0.0001), but not for CLBR per embryo transfer. In terms of classical outcomes of reproductive success such as PR and LBR per ET, no statistically significant difference was found between the MACS and reference groups, again reinforcing the fact that classic metrics may hide subtle contributions when properly measured as per CLBR (Table 3). This study concluded that, although fewer embryos were required to obtain the first live birth after using MACS, it is only a slight improvement compared to the reference group and, thus, the clinical relevance of the additional sperm processing seems low.

## 7. Other Outcomes: Children’s Health

Once the efficiency of the technique has been evaluated, a major concern that needs to be addressed is its safety. This is assessed by evaluating the data available from the pregnancies, deliveries and newborns resulting from transfer of embryos developed after using MACS before ICSI and comparing those to obstetric and perinatal outcomes of standard ICSI cycles.

After studying its effect on reproductive outcomes in a randomized controlled trial, Romany and colleagues [76] compared obstetric and perinatal outcomes of MACS-ICSI cycles with swim-up-ICSI showing that MACS had no clinically relevant adverse effects for either mother or child, presenting no significant differences in rates for premature rupture of membranes (6.1% (0–12.8) in the MACS group vs. 5.9% (0–12.4) in the control group), first trimester bleeding (28.6% (15.9–41.2) vs. 23.5% (11.9–35.1)), gestational diabetes (14.3% (4.5–24.1) vs. 9.8% (1.6–17.9)), gestational anemia (6.1% (0–12.8) vs. 5.9% (0–12.4)) and gestational hypertension (6.1% (0–12.8) vs. 15.7% (5.7–25.7)). Newborns in both groups showed comparable birth weights (2684.10 g (2499.48–2868.72) vs. 2676.12 g (2499.02–2852.21)) and height (48.3 cm (47.1–49.4) vs. 46.5 cm (44.6–48.4), as well as incidence of preterm birth (28.6% (15.9–41.2) vs. 31.3% (18.6–44.0)), very preterm birth (12.2% (3.0–21.4) vs. 10.2% (1.9–18.5)) and admissions into the neonatal intensive care unit (NICU) (13.8% (4.1–23.5) vs. 12.1 (3.1–21.0)) (Table 1). The authors concluded that sperm processing by MACS did not impact obstetric or perinatal outcomes, reporting results comparable to children conceived by standard ICSI in the largest randomized control trial reporting results from live birth with MACS to date.

Consequently, a retrospective study by Gil Juliá and collaborators [77] evaluated a total of 25,356 deliveries (20,439 of which were singleton deliveries) following donor oocyte ICSI cycles, and 19,703 deliveries (15,917 were singleton deliveries) from cycles using autologous oocytes. There were no noteworthy differences between the MACS and reference groups in either of the study populations—donor or patient’s own oocytes—in the main obstetric and perinatal morbidities that would affect the newborn or the mother during pregnancy or delivery (gestational diabetes, gestational hypertension, pre-eclampsia, bleeding, membrane rupture, premature birth, neonatal weight and height or admissions to the NICU). Nonetheless, both study populations showed a significant increase in the rate of gestational anemia when MACS was used for sperm processing compared to the reference (14.03% (10.49–18.22) vs. 9.58% (8.95–10.23), respectively; *p*= 0.01 for donor oocyte cycles; and 19.58% (16.42–23.06) vs. 10.04% (9.41–10.69) using autologous oocytes *p* < 0.001). However, this rate was within the average prevalence for gestational anemia in the standard population. In terms of perinatal outcomes, a statistically significant decline in preterm (9.06% (6.86–11.68) vs. 12.44% (11.98–12.91); *p* = 0.02) and very preterm (1.88% (0.94–3.34) vs. 4.08% (3.81–4.36); *p* = 0.01) birth rates in the MACS group in cycles using donor oocytes was observed (Table 1). This study confirmed the safety of MACS prior to ICSI in cycles using either donor or autologous oocytes from the standpoint of the mother and child’s health during pregnancy and birth. It is also advised that some of these outcomes, such as gestational anemia, are more closely followed to uncover smaller effect sizes.

## 8. Conclusions

To date, the main limitation is the lack of strong-enough evidence of a clear benefit of the non-apoptotic sperm selection via MACS before sperm microinjection. The technique has been widely applied to populations of infertile men without a diagnosis of infertility related to sperm apoptosis; hence, the target population differs between most studies. Moreover, the quality of evidence does not permit the robust definition of the contribution of MACS to standard ART results due to the lack of properly designed RCTs. Nevertheless, we must acknowledge as a limitation that other literature may be available, that has not been considered here, as this was not intended to be a systematic review, although the limited number of papers on the topic make this possibility very unlikely.

The incorrect choice of main outcomes to address the effectiveness of the technique on the end-result of cycles, i.e., the likelihood of achieving a healthy newborn, is often seen in these kinds of publications. Thus, sufficiently powered, well-designed, and executed randomized trials are strongly advised to use cumulative rates per oocyte consumed or per concluded cycle to assess the effect of MACS as a therapeutic option for each specific male candidate. Notwithstanding, there is no solid evidence that raises concern about the possibility of harming the offspring due to its use. In fact, some studies have already addressed the issue of obstetric and perinatal safety of MACS sperm processing.

All in all, the recommendation of using MACS as part of the ART procedure should be very limited and discussed with each couple by clinicians, informing them appropriately about the lack of evidence/definite consensus on their effect on outcomes.

For future research, we may need well-designed and -conducted clinical trials on patients exhibiting increased apoptosis within their sperm, with a sufficient number of cases to be able to statistically proof a clinically meaningful benefit.

## Figures and Tables

**Table 1:** Comparative list of case reports and prospective non-randomized and retrospective studies on magnetic-activated cell sorting (MACS) to fertilize with non-apoptotic sperm in IVF/ICSI cycles. The references are grouped according to the study design and ordered chronologically within the same given type.

Reference (Year)	Study Design	Male Patients’ Inclusion/Exclusion Criteria	Female Patients’ Inclusion/Exclusion Criteria	Intervention (n)	Controls (n)	Control of Bias	Main Outcomes Measured	Effect’s Size and *p* Value
Rawe 2010	Case report	Four years of primary infertility due to male factor. Six cycles of intrauterine inseminations. Thirty-eight-year-old man with bilateral varicocele surgically treated. Previous sperm DNA fragmentation test via TUNEL assay = 31% fragmented spermatozoa (above the normal 20% threshold). Cleaved caspase 3 was present in 8% of spermatozoa (normal expected range 4.8 ± 2.9%)	Four years of primary infertility due to male factor. Six cycles of intrauterine inseminations of a 37-year-old woman.	DGC-MACS-ICSI n = 1	No	Not applicable	Clinical pregnancy	Not applicable
Polak de Fried 2010	Case series	Case 1: asthenoteratozoospermia, abnormal DNA fragmentation (TUNEL 30%) Case 2: Couple with >4 years of primary infertility and recent ICSI failure. Semen with teratozoospermia and abnormal active caspase-3 (16%; when normal <11%).	Case 1: Premature ovarian failure patient with previous fertilization failures. Case 2: Couple with >4 years of primary infertility and recent ICSI failure.	DGC-MACS-ICSI n = 2	No	Not applicable	Progression of the cycle is described (fertilized oocytes, embryo development, transfer and cryopreservation). Ongoing pregnancy Live birth	Not applicable
Herrero 2013	Case report	Cryopreserved spermatozoa with high level of sperm DNA fragmentation from a cancer patient survivor.	Couple with two previous IVF/ICSI failures when the sperm cryopreserved prior to the cancer treatment was used.	DGC-MACS-ICSI n = 1	No	Not applicable	Sperm quality post-MACS Progression of the cycle Live birth	Not applicable
Dirican 2008	Prospective Non-randomized	Oligozoospermia, asthenozoospermia, oligoasthenospermia and/or teratozoospermia.	Primary infertility, maximum baseline FSH of 10 mIU/mL, maximum baseline E2 of 75 pg/mL, ovulatory menstrual cycles, age at the time of screening <35 years old, no uterine abnormalities or communicating hydrosalpinx, no history of low or absent ovarian response during FSH/HMG treatment. Fresh day-3 embryo transfer	MACS-DGC-ICSI n = 122	DGC-ICSI n = 74	Baseline comparisons	Number of 2PN conceptuses Fertilization rate Number of embryos Cleavage rate (%) Number of blastomeres/embryo Fragmentation rate Biochemical pregnancy/transfer (%) Clinical pregnancy/transfer (%) Implantation rate	ns ns 7.7 vs. 7.5 *p* < 0.01 97.2 vs. 88.2 *p* < 0.01 ns ns 61.47 vs. 45.95 *p* < 0.05 48.36 vs. 36.49 *p* < 0.052 ns
Sheikhi 2013	Prospective Non-randomized	Unexplained infertility with previous normal semen parameters: sperm count ≥ 20 million/mL, sperm motility ≥ 30% and normal sperm morphology ≥ 10%.	Couples with unexplained infertility with no obvious male and female infertility factors. Fresh day-3 embryo transfer.	MACS-DGC-ICSI n = 37	DGC-ICSI n = 37	Baseline comparisons Duration of infertility 3.39 vs. 4.59 *p* = 0.002 (years)	Total number of embryos Fertilization rate (%±SD) Cleavage rate Number of 8-grade1 embryos Embryo quality (%±SD) → ratio of 8-grade1 embryos to oocytes injected. Number of blastomeres Pregnancy rate Live birth rate	ns 73.41 ± 22.78 vs. 61.11 ± 24.85 *p* = 0.03 ns ns 45.5 ± 24.82 vs. 34.16 ± 22.37 vs. *p* = 0.049 ns ns ns
García-Ferreira 2015	Prospective Non-randomized	High sperm DNA fragmentation (measured by sperm chromatin dispersion (SCD) using Halosperm kit)	Unclear/Not available. Fresh day-5 transfer	High sperm DNA fragmentation. MACS-DGC-ICSI n = 57 107 embryos were transferred	Normal sperm DNA fragmentation. DGC-ICSI n = 77 146 embryos were transferred	Baseline comparisons Statistically significant (*p* < 0.05) differences between groups in female age and male age. Subanalysis of pregnancy rate, implantation rate and miscarriage rate dividing patients into 3 age groups: <35, 35–39, ≥40.	Total number of fertilized oocytes (2PN) Cleavage rate of embryo at day 3 Number of cells at day 3 Good quality embryos at day 3 Blastocyst development Good quality blastocysts Full blastocyst Expanded blastocyst Hatching blastocyst Total number of embryo transferred/patient Pregnancy rate Implantation rate Single pregnancies Twin pregnancies Miscarriages Biochemical pregnancy rate	ns ns ns ns ns ns ns ns ns ns ns ns ns ns ns ns ns ns
Sanchez-Martín 2017	Retrospective	Volume ≥ 1.5 mL, sperm concentration ≥ 5×106/mL, progressive motility > 15% normal sperm morphology of ≥ 1%. SDF ≥ 30% Excluded: clinical cases with severe male factor infertility.	Inclusion: 6–15 metaphase II (MII) oocytes on the day of oocyte retrieval in autologous cycles. Exclusion: Poor response to ovarian stimulation protocols, polycystic ovary syndrome, adenomyosis, endometriosis grade III and IV, hydrosalpinx, alterations of the endometrium, known genetic alterations and uterine malformations	MACS-DGC-ICSI 30–50% SDF Autologous-ICSI n = 42 Donor-ICSI n = 29 >50% SDF Autologous-ICSI n = 23 Donor-ICSI n = 11	DGC-ICSI 30–50% SDF Autologous-ICSI n = 144 Donor-ICSI n = 40 >50% SDF Autologous-ICSI n = 7 Donor-ICSI n = 9	Baseline comparisons	30–50% SDF (both autologous-ICSI and donor-ICSI) Live birth rate Miscarriage rate <50% SDF (both autologous-ICSI and donor-ICSI) Live birth rate Miscarriage rate Regardless of the SDF Autologous-ICSI Clinical pregnancy (events) Miscarriage (events) Donor-ICSI Clinical pregnancy (events) Miscarriage (events)	ns ns ns ns overall *p* = 0.0436 26 vs. 93 0 vs. 7 26 vs. 32 0 vs. 3
Pacheco 2020	Retrospective	Couples using both own and donor oocytes. >20% sperm DNA fragmentation measured by TUNEL assay. Exclusion: seminal infection, orchitis/epididymitis, AZF microdeletions, altered karyotype, sperm aneuploidies determined by fluorescent in situ hybridization (FISH) analysis, and patients with systemic diseases or history of cryptorchidism.	Couples using both own and donor oocytes.	DGC-MACS-ICSI n = 366 PGT-A ICSI n = 126 Autologous ova n = 121 Donated ova n = 119	DGC-ICSI n = 358 PGT-A ICSI n = 116 Autologous ova n = 120 Donated ova n = 122	Baseline comparisons Subanalysis separating three subpopulations: PGT-A Autologous ova Donor oocytes	ALL Fertilization rate Pregnancy rate (%) Miscarriage rate (%) Livebirth rate (%) PGT-A SDF Fertilization rate Pregnancy rate Miscarriage rate Live-birth rate AUTOLOGOUS SDF Fertilization rate Pregnancy rate Miscarriage rate (%) Live-birth rate (%) DONATED SDF Fertilization rate Pregnancy rate (%) Miscarriage rate Live-birth rate (%)	ns 60.7 vs. 51.5 *p* = 0.014 14.7 vs. 20.6 *p* = 0.034 47.4 vs. 31.2 *p* = 0.001 ns ns ns ns ns ns ns ns 11.3 vs. 25.5 *p* = 0.005 40.9 vs. 24.6 *p* = 0.03 ns ns 69.6 vs. 53.9 *p* = 0.013 ns 51.8 vs. 29.4 *p* = 0.03
Gil Juliá 2023	Retrospective	Unselected males	Couples undergoing ICSI cycles using either donor or autologous oocytes	MACS-ICSI Various capacitation methods considered Cycles using donor oocytes n = 705 deliveries, 587 singleton deliveries. Cycles using autologous oocytes n = 880 deliveries, 746 singleton deliveries.	Standard ICSI Cycles using donor oocytes n = 24,651 deliveries, 19,852 singleton deliveries. Cycles using autologous oocytes n = 18,193 deliveries, 15,171 singleton deliveries.	Baseline comparisons Multivariant analysis	Type of birth Cesarean section/Vaginal birth Type of cesarean section Scheduled/Intrapartum/Emergency Type of vaginal birth Spontaneous/Induced Gestational diabetes Gestational anemia (% (95%CI)) Gestational hypertension Proteinuria Pre-eclampsia Bleeding Amniocentesis Bleeding during the 2nd or 3rd trimester Membrane rupture Week of membrane rupture Puerperium pathology Weight Low birthweight (% (95%CI)) Very low birthweight (% (95%CI)) Neonatal length Head circumference Gestational age (weeks. mean (95%CI)) Premature birth (% (95%CI)) Very premature birth (% (95%CI)) Sex of the newborn Apgar score at 1min Apgar score at 5 min (mean (95%CI)) Apgar score at 10 min Admissions to the NICU	ns S ns ns ns 14.03 (10.49–18.22) vs. 9.58 (8.95–10.23) *p* = 0.004 (donor), 19.58 16.42–23.06) vs. 10.04 (9.41–10.69) *p* < 0.001 (autologous) ns ns ns ns ns ns ns ns ns ns 11.80 (8.56–15.72) vs. 10.32 (9.82–10.84) *p* = 0.03 (donor) 0.42 (0.05–1.50) vs. 0.99 (0.82–1.19) *p* < 0.001 (autologous) ns ns 8.37 (6.47–10.60) vs. 7.83 7.40–8.27) *p* = 0.009 (autologous) 9.06 (6.86–11.68) vs. 12.44 (11.98–12.91) *p* = 0.04 (donor) 1.88 (0.94–3.34) vs. 4.08 (3.81–4.36) *p* = 0.01 (donor) ns ns 9.67 (9.59–9.75) vs. 9.84 (9.72–9.76) *p* = 0.02 (autologous) ns ns

DGC = density gradient centrifugation. DFI = DNA fragmentation index. ns = not significant. SD = standard deviation. 95%CI = 95% confidence interval.

**Table 2:** Comparative list of prospective randomized studies and randomized clinical trials (RCTs) evaluating the impact of magnetic-activated cell sorting (MACS) on IVF/ICSI cycle outcomes. The references are grouped according to the study design and ordered chronologically within the same given type.

Reference (Year)	Study Design	Male Patients’ Inclusion/Exclusion Criteria	Female Patients’ Inclusion/Exclusion Criteria	Intervention (n)	Controls (n)	Control of Bias	Main Outcomes Measured	Effect’s Size and *p* Value
Stimpfel 2018	Prospective Sibling oocytes Randomized	Couples who were recommended ICSI due to male factor—teratozoospermia (<15% normal morphology according to the strict Kruger criteria).	Women not older than 36, with a normal ovarian response to controlled ovarian hyperstimulation (≥6 mature oocytes after ultrasound retrieval). Exclusion: previous births or pregnancies.	For each couple, one half of the mature oocytes was fertilized with DGC-MACS-Swim up-ICSI n = 127 oocytes	The other half was fertilized with DGC-ICSI n = 133 oocytes	Sibling oocytes design Subanalysis according to the female patient’s age: ≤30 vs. ≥31.	Fertilized oocytes Cleaved embryos Good quality day 3 Fair quality day 3 Poor quality day 3 Number of embryos cultured until the day 5 Number of blastocysts Good quality blastocyst Fair quality blastocyst 8 Poor quality blastocyst Number of frozen blastocysts Number of cycles with blastocyst freezing Implantation rate Number of pregnancies Number of deliveries For women ≥31 years old: Good quality blastocysts (%)	ns ns ns ns ns ns ns ns ns ns ns ns ns ns ns ns ns 75.0 vs. 33.3 (*p* < 0.05)
Salehi Novin 2022	Prospective Sibling oocytes Randomized	Severe male factor infertility DFI >30% measured by SDF assay (Halosperm)	-	MACS-DGC n = 60 102 oocytes	DGC n = 60 86 oocytes	Randomization (Sibling sperm samples, sibling oocytes) and baseline comparisons	DFI—means not stated Morphology—means not stated Total motility—means not stated Fertilization rate Top-quality day-3 embryos (%±SD) Blastocyst rate (%) PLCZ1 gene expression—values not stated	*p* = 0.000 *p* = 0.001 *p* = 0.028 ns 85 ± 0.57 vs. 63.23 ± 0.44 *p* = 0.038 69.69 vs. 48.00 *p* < 0.001 *p* = 0.046
Romany 2014	RCT	Unselected men. >10% motile sperm in raw sample, >1 million motile sperm after swim-up.	First ICSI with donor oocytes. Fresh day-3 embryo transfer. Female partners: 30–45 years old, body mass index (BMI) < 30 kg/m^2^, confirmed absence of reported uterine pathology, and no clinical history of recurrent miscarriage.	Swim-up-MACS-ICSI n= 123	Swim-up-ICSI n = 114	Randomization Triple-blind Baseline characteristics Per embryo transferred Per intention to treat	Fertilization rate (%) Good-quality embryos (%) Implantation rate (%) Positive beta-hCG (%) Live-birth rate (%) Miscarriage rate (%)	ns ns ns ns ns ns
Troya 2015	RCT	Unselected males. Normal sperm concentration according to the WHO 2010 criterion.	Exclusion: Endometriosis.	DGC-PICSI n = 45 or DGC-MACS-ICSI n = 33	DGC-ICSI n = 55	Randomization and baseline characteristics	Fertilization rate (% (95%CI)) Number of day-3 embryos Number of freezing embryos Clinical pregnancy rate (%) Biochemical pregnancy rate Pregnancy loss rate	70.15 (63.98–76.33)/80.28 (73.74–86.81)/78.97 (74.37–83.57) *p* < 0.05 ns ns 40.40/58.10/27.30 *p* < 0.05 ns ns
Romany 2017	RCT	Unselected men. >10% motile sperm in raw sample, >1 million motile sperm after swim-up.	First ICSI with donor oocytes. Fresh day-3 embryo transfer. Female partners: 30–45 years old, body mass index (BMI) < 30 kg/m^2^, confirmed absence of reported uterine pathology, and no clinical history of recurrent miscarriage.	Swim-up-MACS-ICSI n= 65 newborns	Swim-up-ICSI n = 66 newborns	Randomization Triple-blind	1st trimester bleeding Invasive procedures Gestational anemia Gestational cholestasis Gestational diabetes 2nd and 3rd trimester bleeding Premature rupture of membranes earlier than 37 weeks Urinary tract infection Weeks at delivery Preterm birth Very preterm birth Cesarean section Female newborns Birth weight Birth weight female Birth weight male Low birthweight Very low birthweight Neonatal height Apgar score at 1 min Apgar score at 5 min Apgar score at 10 min Birth defects Major malformations Minor malformations Admissions to neonatal intensive care unit (NICU) Days at NICU	ns ns ns ns ns ns ns ns ns ns ns ns ns ns ns ns ns ns ns ns ns ns ns ns ns ns ns
Ziarati 2019	RCT	At least two semen parameters below WHO 2010 criteria. Exclusion: seminal infection, history of cryptorchidism sperm, autoantibodies, orchitis, or those that have systemic diseases or endocrine disorders.	Exclusion: >42 years-old, <6 matured oocytes retrieved, poor quality oocyte.	MACS-DGC-ICSI procedure n = 29	DGC-ICSI n = 33	Randomization and baseline parameters	Fertilization rate Embryo score A (%±SD) Embryo score B Embryo score C Mean of embryo score Mean embryo score of transferred embryos Clinical pregnancy (%) Implantation rate (%)	ns 35.85 ± 6.58 vs. 20.00 ± 3.93 *p* = 0.04 ns ns ns ns 54.54 vs. 24.24 *p* < 0.01 36.30 vs. 15.70 *p* < 0.02
Hasanen 2020	RCT	Abnormal sperm SDF (≥20.3%) Total progressive motile sperm count ≥ 1 million Exclusion: Leukocytospermia, varicocele, known genetic disorder, or any known factors that affect ovarian stimulation or embryo implantation.	18–35 years old ≥5 mature oocytes retrieved	DGC-MACS-ICSI n = 196	DGC-PICSI n = 200	Baseline comparisons Subanalysis according to male age: ≤35 years 36–41 years >41 years And female age: <30 years 30–35 years	Overall Ongoing pregnancy Cleavage rate Blastulation rate Good-quality blastulation rate Clinical pregnancy rate Implantation rate Subanalysis acc to male age Subanalysis acc to female age Ongoing pregnancy (%) <30 y.o Good-quality blastulation rate (%±SD) <30 y.o Clinical pregnancy rate (%) <30 y.o Implantation rate (%) 30–35 y.o	ns ns ns ns ns ns all ns 69.5 vs. 51.3 *p* = 0.01 71.1 ± 25.4 vs. 62.9 ± 24.7 *p* = 0.03 73.9 vs. 58.3 *p* = 0.03 33.3 vs. 46 *p* = 0.05
González-Ravina 2022	RCT	Donor sperm intrauterine inseminations (D-IUI).	At least one permeable fallopian tube and normal ovulation.	DGC-MACS-IUI n = 90	DGC-IUI n = 91	Randomization and baseline comparisons	Clinical pregnancy rate Live-birth rate Miscarriage rate	ns ns ns
Gil 2013	Metanalysis—five prospective randomized trials: 499 patients.	Unselected males	Unselected	MACS	Standard semen sample preparation	Metanalysis	Pregnancy rate Implantation rate Miscarriage rate	*p* = 0.004 RR = 1.50 (1.14–1.98) ns RR = 1.03 (0.80–1.31) ns RR = 2.00 (0.19–20.90)
Lepine 2019	Metanalysis—RCTs		None	MACS-ICSI	Standard ICSI	Metanalysis	Live birth rate (1 RCT) Clinical pregnancy rate (3 RCTs) Miscarriage rate (2 RCTs)	RR 1.95 (0.89–4.29) RR 1.05 (0.84–1.31) RR 0.51 (0.09–2.82) Uncertain of the effect of MACS on those outcomes. Quality of evidence was very low.

DGC = density gradient centrifugation. DFI = DNA fragmentation index. ns = not significant. SD = standard deviation. 95%CI = 95% confidence interval. RR = risk ratio.

**Table 3:** Summary of papers that assess the effect of magnetic-activated cell sorting (MACS) in cumulative live birth rates (CLBR).

Reference (Year)	Study Design	Male Patients’ Inclusion/Exclusion Criteria	Female Patients’ Inclusion/Exclusion Criteria	Intervention (n)	Controls (n)	Control of Bias	Main Outcomes Measured	Effect’s Size and *p* Value
Gil Juliá 2021	Retrospective	Unselected males	Couples using the female patients’ own oocytes (autologous)	MACS-ICSI Various capacitation methods considered	Standard ICSI	Baseline comparisons Multivariant analysis Subanalysis of live birth rate according to performance or not of PGT-A	Per transfer Biochemical pregnancy rate Clinical pregnancy rate Ongoing pregnancy rate Live birth rate Clinical miscarriage rate Per cycle Live birth rate Cumulative live birth rates Per MII oocyte used Per embryo replaced Per embryo transfer	ns ns ns ns ns ns ns *p* < 0.001 *p* = 0.009
Gil Juliá 2022	Retrospective	Unselected males	Couples using donor oocytes.	MACS-ICSI Various capacitation methods considered	Standard ICSI	Baseline comparisons Multivariant analysis	Per transfer Biochemical pregnancy rate Clinical pregnancy rate Ongoing pregnancy rate Live birth rate Clinical miscarriage rate Per cycle Live birth rate Cumulative live birth rates Per MII oocyte used Per embryo replaced Per embryo transfer	ns ns ns ns ns ns *p* < 0.001 *p* < 0.001 ns

DGC = density gradient centrifugation. DFI = DNA fragmentation index. ns = not significant. SD = standard deviation. 95%CI = 95% confidence interval. RCT = randomized controlled trial. RR = risk ratio.

## Data Availability

No new data were created or analyzed in this study. Data sharing is not applicable to this article.
